# Relationship between abdominal circumference and the incidence of hyperuricemia in the general Japanese population

**DOI:** 10.1038/s41598-024-55008-6

**Published:** 2024-02-25

**Authors:** Kazumi Kawano, Tamami Ueno, Toshiki Maeda, Chihiro Nohara, Kaori Maki, Kazuyo Iwanaga, Akiko Morinaga, Shunsuke Funakoshi, Makiko Abe, Atsushi Satoh, Miki Kawazoe, Chikara Yoshimura, Koji Takahashi, Kazuhiro Tada, Kenji Ito, Tetsuhiko Yasuno, Shigeaki Mukobara, Daiji Kawanami, Kosuke Masutani, Hisatomi Arima

**Affiliations:** 1 Department of Preventive Medicine and Public Health, Fukuoka University, 7-45-1 Nanakuma, Jonan-ku, Fukuoka City, Japan; 2 Faculty of Medicine, School of Nursing, Fukuoka University, Fukuoka, Japan; 3 Department of Pediatrics, Yokohama City University, Yokohama, Japan; 4 Department of Nursing, Faculty of Medicine, Kurume University, Fukuoka, Japan; 5 Department of Medical Laboratory Science, Faculty of Health Science, Junshin Gakuen University, Fukuoka, Japan; 6 Division of Nephrology and Rheumatology, Department of Internal Medicine, Faculty of Medicine, Fukuoka University, Fukuoka, Japan; 7 Department of Internal Medicine, Nagasaki Prefecture Iki Hospital, Nagasaki, Japan; 8 Department of Endocrinology and Diabetes Mellitus, Faculty of Medicine, Fukuoka University, Fukuoka, Japan

**Keywords:** Health care, Risk factors

## Abstract

In this study, we aimed to separately evaluate the relationship between waist circumference and the incidence of hyperuricemia in men and women in the general Japanese population. We performed a population-based longitudinal study using data from the annual health examination of residents of Iki City, Japan. A total of 5567 participants without hyperuricemia at baseline were included in the analysis. The men and women were placed into groups according to the tertile of waist circumference. The outcome was incident hyperuricemia (uric acid > 416 µmol/L [7.0 mg/dL]). The relationship between waist circumference and the incidence of hyperuricemia was investigated using Cox proportional hazards models. During the follow-up period, hyperuricemia developed in 697 people (551 men and 146 women). The incidence (per 1000 person-years) of hyperuricemia increased with increasing waist circumference in the men (34.9 for tertile 1, 49.9 for tertile 2 and 63.3 for tertile 3; *P*_trend_ < 0.001) and women (5.5 for tertile 1, 6.3 for tertile 2 and 11.9 for tertile 3; *P*_trend_ < 0.001). Significant associations were identified after adjustment for potential confounders (men: *P*_trend_ < 0.001; women: *P*_trend_ = 0.014). In conclusion, both men and women with larger waist circumferences were at higher risks of subsequent hyperuricemia.

## Introduction

Hyperuricemia is a substantial risk factor for gouty arthritis owing to urate deposition and ureteral stones^1^. In recent years, hyperuricemia has also been reported to be associated with higher risks of chronic kidney disease (CKD), cardiovascular disease and all-cause mortality^2^. The prevention of hyperuricemia and subsequent gout, ureteral stones, CKD, cardiovascular disease and death requires an approach based on the latest information regarding the risk factors.

The links of fat distribution with subsequent gout and urinary tract stone disease has been recognised since the 1950s^3^. In addition to overall obesity, abdominal obesity has been reported to be strongly associated with metabolic disorders and greater risks of various diseases^4–6^. Although the body mass index (BMI) is commonly used in clinical practice, it cannot distinguish fat mass from lean mass and does not provide information regarding fat distribution. Current evidence regarding the relationship between obesity and hyperuricemia is derived mainly from observational studies in which BMI was used; Therefore, the link between abdominal obesity (which closely correlates with waist circumference^7,8^) and uric acid metabolism has not been well characterised. Two previous cross-sectional studies have shown significant associations between waist circumference and hyperuricemia in both men and women^9,10^. In addition, a longitudinal study of 2,895 middle-aged Chinese people showed an association between large waist circumference (≥ 95 cm in men and ≥ 85 cm in women) and a high incidence of hyperuricemia, although the dose-dependency and sex predisposition of the relationship were not evaluated^11^. Therefore, there is uncertainty surrounding the longitudinal, dose-dependent, and sex-specific relationships between waist circumference and the incidence of hyperuricemia, particularly in Japanese people.

In the present study, we aimed to clarify the relationships between waist circumference and the subsequent development of hyperuricemia in men and women in the general Japanese population.

## Results

The participants were placed into sex-specific groups according to their waist circumference: tertile 1 (≤ 81.0 cm), tertile 2 (81.1–88.0 cm) and tertile 3 (> 88.0 cm) for men; and tertile 1 (≤ 78.0 cm), tertile 2 (78.1–86.5 cm) and tertile 3 (> 86.5 cm) for women.

Tables 1 and 2 present the baseline characteristics of the participants, according to tertile of waist circumference, for men (Table 1) and women (Table 2). Both men and women with larger waist circumferences were more likely to have hypertension, diabetes and dyslipidaemia.

**Table 1:** Baseline characteristics according to tertile of waist circumference among men.

	Waist circumference (cm)			*p*-value for the trend
	Tertile 1 ≤ 81.0 cm N = 793	Tertile 2 81.1ー88.0 cm N = 772	Tertile 3 > 88.0 cm N = 1072	
Age, mean (SD), years	58.9 (11.2)	60.9 (9.8)	61.5 (9.4)	< 0.001
Body mass index, mean (SD), kg/m^2^	21.1 (1.9)	23.6 (1.7)	27.0 (2.6)	< 0.001
Obesity*, N (%)	18 (2.3)	158 (20.5)	607 (79.2)	< 0.001
Uric acid, mean (SD), µmol/L	315.6 (57.6)	331.5 (57.6)	332.3 (58.7)	< 0.001
Systolic blood pressure, mean (SD), mmHg	126.4 (17.9)	131.3 (17.5)	135.3 (17.6)	< 0.001
Diastolic blood pressure, means(SD), mmHg	74.0 (11.1)	76.2 (10.6)	78.9 (10.8)	< 0.001
Hypertension**, N (%)	274 (34.6)	405 (52.5)	483 (63.1)	< 0.001
HbA1c, mean (SD)	5.4 (0.7)	5.5 (0.8)	5.7 (0.9)	< 0.001
Diabetes mellitus***, N (%)	70 (8.8)	107 (13.9)	154 (20.2)	< 0.001
High-density lipoprotein cholesterol, mean (SD), mmol/L	1.7 (0.4)	1.5 (0.4)	1.4 (0.4)	< 0.001
Low-density lipoprotein cholesterol, mean (SD), mmol/L	2.9 (0.7)	3.0 (0.8)	3.1 (0.8)	< 0.001
Triglyceride, mean (SD), mmol/L	1.1 (0.6)	1.4 (0.8)	1.7 (1.2)	< 0.001
Dyslipidemia****, N (%)	213 (26.8)	331 (42.9)	407 (53.1)	< 0.001
Estimated glomerular filtration rate, mean (SD), mL/min/1.73m^2^	78.6 (15.2)	76.5 (15.6)	74.6 (16.1)	< 0.001
Urine protein, N (%)	49 (6.2)	47 (6.1)	72 (9.4)	0.016
Chronic kidney disease*****, N (%)	101 (12.8)	121 (15.8)	166 (21.8)	< 0.001
Smoking, N (%)	306 (38.6)	249 (32.3)	232 (30.3)	< 0.001
Current daily alcohol intake, N (%)	302 (40.3)	332 (47.0)	306 (44.7)	0.087
Regular exercise, N (%)	236 (30.7)	205 (27.8)	208 (28.6)	0.360

*Body mass index of ≥ 25 kg/m^2^. **BP ≥ 140/90 mmHg or us of blood pressure lowering medication. ***Fasting blood glucose concentration ≥ 7.0 mmol/L, Non-fasting blood glucose concentration ≥ 11.1 mmol/L, HbA1c values ≥ 6.5%, or use of glucose-lowering therapy. ****LDH cholesterol concentrations ≥ 3.62 mmol/L, HDL cholesterol concentrations < 1.03 mmol/L, triglyceride concentrations ≥ 1.69 mmol/L, or use of lipid-lowering medication. *****eGFR < 60 mL/min/1.73m^2^ or urine protein ≥ 1.

**Table 2:** Baseline characteristics according to tertile of waist circumference among women.

	Waist circumference (cm)			*p*-value for the trend
	Tertile 1 ≤ 78.0 cm N = 1081	Tertile 2 78.1ー86.5 cm N = 1083	Tertile 3 > 86.5 cm N = 1072	
Age, mean (SD), years	57.7 (11.8)	61.2 (9.1)	62.8 (8.4)	< 0.001
Body mass index, mean (SD), kg/m^2^	20.5 (2.0)	23.0 (2.1)	26.9 (3.3)	< 0.001
Obesity*, N (%)	18 (1.7)	200 (18.5)	754 (70.3)	< 0.001
Uric acid, mean (SD), µmol/L	243.3 (54.4)	263.8 (55.5)	279.5 (57.2)	< 0.001
Systolic blood pressure, mean (SD), mmHg	123.5 (19.0)	128.8 (18.4)	133.9 (18.7)	< 0.001
Diastolic blood pressure, mean (SD), mmHg	71.3 (10.8)	74.0 (10.5)	75.8 (10.6)	< 0.001
Hypertension**, N (%)	324 (30.0)	471 (43.5)	639 (59.6)	< 0.001
HbA1c, mean (SD)	5.3 (0.5)	5.4 (0.6)	5.6 (0.8)	< 0.001
Diabetes mellitus***, N (%)	43 (4.0)	70 (6.5)	146 (13.6)	< 0.001
High-density lipoprotein cholesterol, mean (SD), mmol/L	1.8 (0.4)	1.7 (0.5)	1.6 (0.4)	< 0.001
Low-density lipoprotein cholesterol, mean (SD), mmol/L	3.1 (0.8)	3.4 (0.8)	3.4 (0.8)	< 0.001
Triglyceride, mean (SD), mmol/L	1.0 (0.5)	1.2 (0.8)	1.4 (0.8)	< 0.001
Dyslipidemia****, N (%)	325 (30.1)	478 (44.1)	564 (52.6)	< 0.001
Estimated glomerular filtration rate, mean (SD), mL/min/1.73m^2^	75.5 (14.3)	73.7 (14.8)	72.9 (15.0)	< 0.001
Urine protein, N (%)	30 (2.8)	30 (2.8)	53 (5.0)	0.006
Chronic kidney disease*****, N (%)	136 (12.7)	168 (15.5)	193 (18.1)	< 0.001
Smoking, N (%)	107 (9.9)	60 (5.5)	58 (5.4)	< 0.001
Current daily alcohol intake, N (%)	81 (7.9)	43 (4.3)	32 (3.3)	< 0.001
Regular exercise, N (%)	246 (24.0)	267 (25.8)	278 (27.3)	0.085

*Body mass index of ≥ 25 kg/m^2^. **BP ≥ 140/90 mmHg or us of blood pressure lowering medication. ***Fasting blood glucose concentration ≥ 7.0 mmol/L, Non-fasting blood glucose concentration ≥ 11.1 mmol/L, HbA1c values ≥ 6.5%, or use of glucose-lowering therapy. ****LDH cholesterol concentrations ≥ 3.62 mmol/L, HDL cholesterol concentrations < 1.03 mmol/L, triglyceride concentrations ≥ 1.69 mmol/L, or use of lipid-lowering medication. *****eGFR < 60 mL/min/1.73m^2^ or urine protein ≥ 1.

During follow-up (mean 5.4 years), 697 people (551 men and 146 women) developed hyperuricemia. Table 3 shows the risk of hyperuricemia in each group of men and women. The incidence of hyperuricemia (per 1000 person-years) was higher in men with larger waist circumferences: 34.9 for tertile 1, 49.9 for tertile 2 and 63.3 for tertile 3 (*P*_trend_ < 0.001). This relationship was also significant after adjustment for age, hypertension, diabetes, dyslipidaemia, CKD, current smoking, current alcohol consumption and exercise status: the multivariable-adjusted hazard ratios were 1.25 (95% confidence interval [95% CI] 0.99–1.57) for tertile 2 and 1.49 (95% CI 1.18–1.89) for tertile 3, compared with tertile 1 (*P*_trend_ < 0.001). Among men, mediation analysis revealed significant pure indirect effects of hypertension (*P* = 0.007) but similar results were obtained from sensitivity analysis excluding hypertension from covariates: the multivariable-adjusted hazard ratios were 1.31 (95% CI 1.04–1.65) for tertile 2 and 1.62 (95% CI 1.29–2.04) for tertile 3, compared with tertile 1 (*P*_trend_ < 0.001). There were no significant mediation effects of other confounding factors. Similar results were also obtained for women, the incidences (per 1,000 person-years) were 5.5 for tertile 1, 6.3 for tertile 2 and 11.9 for tertile 3 (*P*_trend_ < 0.001); and the adjusted hazard ratios were 0.93 (95% CI 0.56–1.54) for tertile 2 and 1.67 (95% CI 1.05–2.66) for tertile 3, compared with tertile 1 (*P*_trend_ = 0.014). Among women, there were no significant mediation effects of confounding factors. There were no clear differences in the multivariable-adjusted associations between the tertile groups of waist circumference and the incidence of hyperuricemia in men and women (*P* = 0.136 for the interaction).

**Table 3:** Risk of hyperuricemia according to tertiles of the waist circumference among men and women.

	Waist circumference*			*p* value for trend
	Tertile 1	Tertile 2	Tertile 3	
Men	N=793	N=772	N=766	
N of events/person-years	141/4040	191/3824	219/3460	
Incidence rate (per 1000 person-years)	34.9	49.9	63.3	
Crude hazard ratio (95% confidence interval)	1.00 (Reference)	1.44 (1.16–1.79)	1.80 (1.46–2.22)	<0.001
Adjusted hazard ratio **(95% confidence interval)	1.00 (Reference)	1.25 (0.99–1.57)	1.49 (1.18–1.89)	<0.001
Women	N=1081	N=1083	N=1072	
N of events/person-years	35/6307	41/6488	70/5888	
Incidence rate (per 1000 person-years)	5.5	6.3	11.9	
Crude hazard ratio (95% confidence interval)	1.00 (Reference)	1.14 (0.73–1.79)	2.13 (1.42–3.19)	<0.001
Adjusted hazard ratio **(95% confidence interval)	1.00 (Reference)	0.93 (0.56–1.54)	1.67 (1.05–2.66)	0.014

*Tertile 1: ≤ 81.0 cm for men and ≤ 78.0 cm for women; Tertile 2: 81.1–88.0 cm for men and 78.1–86.5 cm for women; Tertile 3: > 88.0 cm for men and > 86.5 cm for women.

**Adjusted for age, hypertension, diabetes mellitus, dyslipidemia, chronic kidney disease, smoking, current daily alcohol intake, and regular exercise.

Tables 4 and 5 show the multivariable-adjusted hazard ratios associated with waist circumference for the incidence of hyperuricemia in subgroups of men (Table 4) and women (Table 5). Closer associations between waist circumference and the incidence of hyperuricemia were obtained for men aged ≥ 65 years (hazard ratio 2.19, 95% CI 1.46–3.29 for tertile 3 *vs*. tertile 1) than for men aged < 65 years (hazard ratio 1.26, 95% CI 0.93–1.69, *P* = 0.015 for the interaction). There were no other significant differences in the relationships of waist circumference with hyperuricemia in subgroups defined by hypertension, diabetes, dyslipidaemia, CKD, smoking habits, alcohol consumption or exercise status for the men or the women (interactions all *P* > 0.1).

**Table 4:** Multivariable-adjusted hazard ratios of waist circumference for the incidence of hyperuricemia in subgroups among men.

	Waist circumference			p value for interaction
	Tertile 1	Tertile 2	Tertile 3	
	≤ 81.0 cm	81.1–88.0 cm	> 88.0 cm	
	N = 793	N = 772	N = 766	
Age				
≥ 65 years	1(reference)	1.66 (1.10–2.50)	2.19 (1.46–3.29)	0.015
< 65 years	1(reference)	1.09 (0.82–1.46)	1.26 (0.93–1.69)	
Hypertension				
Yes	1(reference)	1.34 (0.95–1.89)	1.59 (1.14–2.21)	0.888
No	1(reference)	1.13 (0.81–1.58)	1.38 (0.98–1.95)	
Diabetes mellitus				
Yes	1(reference)	1.12 (0.50–2.53)	1.43 (0.66–3.11)	0.881
No	1(reference)	1.28 (1.00–1.64)	1.54 (1.20–1.97)	
Dyslipidemia				
Yes	1(reference)	1.06 (0.73–1.55)	1.36 (0.94–1.96)	0.511
No	1(reference)	1.41 (1.05–1.90)	1.60 (1.17–2.18)	
Chronic kidney disease				
Yes	1(reference)	1.25 (0.72–2.20)	1.54 (0.89–2.64)	0.997
No	1(reference)	1.27 (0.98–1.64)	1.52 (1.17–1.98)	
Smoking				
Yes	1(reference)	1.29 (0.90–1.85)	1.41 (0.96–2.08)	0.207
No	1(reference)	1.31 (0.96–1.78)	1.64 (1.21–2.23)	
Current daily alcohol intake				
Yes	1(reference)	1.24 (0.90–1.70)	1.34 (0.97–1.85)	0.426
No	1(reference)	1.25 (0.88–1.78)	1.75 (1.24–2.47)	
Regular exercise				
Yes	1(reference)	1.55 (0.97–2.49)	1.51 (0.94–2.42)	0.432
No	1(reference)	1.15 (0.88–1.51)	1.47 (1.12–1.93)	

Values are hazard ratios (95%confidence intervals) adjusted for age (except for subgroup analysis by age), hypertension (except for subgroup analysis by hypertension), diabetes mellitus (except for subgroup analysis by diabetes mellitus), dyslipidemia (except for subgroup analysis by dyslipidemia), chronic kidney disease (except for subgroup analysis by chronic kidney disease), smoking (except for subgroup analysis by current smoking), current alcohol intake (except for subgroup analysis by current alcohol intake), and regular exercise (except for subgroup analysis by regular exercise).

**Table 5:** Multivariable-adjusted hazard ratios of waist circumference for the incidence of hyperuricemia in subgroups among women.

	Waist circumference			*p* value for interaction
	Tertile 1	Tertile 2	Tertile 3	
	≤ 78.0 cm	78.1ー86.5 cm	> 86.5 cm	
	N = 1081	N = 1083	N = 1072	
Age				
≥ 65 years	1 (reference)	1.18 (0.51–2.72)	1.89 (0.89–4.03)	0.756
< 65 years	1 (reference)	0.84 (0.44–1.61)	1.58 (0.85–2.92)	
Hypertension				
Yes	1 (reference)	0.86 (0.41–1.84)	1.75 (0.91–3.36)	0.853
No	1 (reference)	0.99 (0.50–1.96)	1.56 (0.78–3.13)	
Diabetes mellitus				
Yes	1 (reference)	1.96 (0.21–17.91)	3.73 (0.47–29.65)	0.894
No	1 (reference)	0.88 (0.52–1.49)	1.59 (0.98–2.58)	
Dyslipidemia				
Yes	1 (reference)	1.15 (0.55–2.42)	2.01 (1.01–4.00)	0.859
No	1 (reference)	0.80 (0.39–1.65)	1.50 (0.79–2.87)	
Chronic kidney disease				
Yes	1 (reference)	0.64 (0.24–1.72)	1.26 (0.53–3.03)	0.542
No	1 (reference)	1.10 (0.61–1.99)	1.84 (1.06–3.20)	
Smoking				
Yes	1 (reference)	4.22 (0.74–23.94)	2.52 (0.48–13.24)	0.706
No	1 (reference)	0.86 (0.51–1.47)	1.59 (0.98–2.59)	
Current daily alcohol intake				
Yes	1 (reference)	4.75 (0.67–33.77)	3.72 (0.42–33.10)	0.680
No	1 (reference)	0.87 (0.51–1.48)	1.61 (0.99–2.61)	
Regular exercise				
Yes	1 (reference)	0.34 (0.12–0.99)	1.16 (0.53–2.56)	0.130
No	1 (reference)	1.30 (0.71–2.37)	1.93 (1.08–3.46)	

Values are hazard ratios (95%confidence intervals) adjusted for age (except for subgroup analysis by age), hypertension (except for subgroup analysis by hypertension), diabetes mellitus (except for subgroup analysis by diabetes mellitus), dyslipidemia (except for subgroup analysis by dyslipidemia), chronic kidney disease (except for subgroup analysis by chronic kidney disease), smoking (except for subgroup analysis by current smoking), current alcohol intake (except for subgroup analysis by current alcohol intake), and regular exercise (except for subgroup analysis by regular exercise).

## Discussion

In the present observational study of the general Japanese population, waist circumference was shown to be associated with subsequent hyperuricemia in both men and women. These associations remained significant after adjustment for the effects of the potential confounders age, hypertension, diabetes, dyslipidaemia, CKD, smoking, daily alcohol consumption and exercise status. There were no clear differences in the relationships of waist circumference with hyperuricemia in the subgroups, although there was a closer association for men aged ≥ 65 years.

In most previous studies, the relationship between obesity and hyperuricemia was investigated using BMI^12–15^. A cross-sectional study of 5591 Korean adults showed significant associations between waist circumference and hyperuricemia in both men and women^9^. A cross-sectional study of 33,498 Japanese people also showed significant increments in the prevalence of hyperuricemia with increases in waist circumference in both men and women^10^. There have been few longitudinal studies, but a cohort study of 2895 middle-aged Chinese adults showed that the odds ratio of hyperuricemia for the highest quartile of waist circumference was 2.2 times higher than that for the lowest quartile^11^. To the best of our knowledge, the present study is the first longitudinal study of the general Japanese population to confirm the findings of previous studies, and has revealed clear associations between waist circumference and the incidence of hyperuricemia in both men and women in Japan.

In the subgroup analysis performed in the present study, a closer association was identified between waist circumference and the incidence of hyperuricemia in men aged ≥ 65 years than in those aged < 65 years (*P* = 0.015 for the interaction). Although this might have been a chance finding, the interaction might be attributable to the higher risk of hyperuricemia that is present in older individuals^16,17^. Further investigation of the role of the interaction between age and abdominal obesity with respect to the development of hyperuricemia is required.

The pathophysiological mechanisms underlying the association between obesity and the development of hyperuricemia are complex and not fully understood. One possible mechanism is that insulin resistance and the subsequent hyperinsulinemia, which are closely associated with central obesity, have effects on the proximal tubules, increasing the reabsorption of sodium-bound uric acid^18–20^. Another is that the expansion of adipose tissue depots and the development of central obesity result in hyperuricemia through excessive uric acid production^21^, because adipose tissue expresses xanthine oxidoreductase (XOR), and therefore generates uric acid^22,23^. The association between waist circumference and hyperuricemia might also be mediated by lifestyle-related diseases, such as high blood pressure, impaired glucose tolerance and dyslipidaemia^10^.

Although the present study was a large-scale longitudinal study of the general Japanese population, it had several limitations. First, because it was a retrospective study, the results of the analysis may have been affected by selection bias. Second, we recruited people who attended health examinations, and therefore they were probably more likely to be aware of healthy lifestyle habits than those who did not attend such examinations. Third, the exact date of onset of hyperuricemia could not be determined, because it was diagnosed at annual physical examinations. Fourth, information regarding the use of urate-lowering medications was not available. Finally, the findings were based on a single measurement of waist circumference, which may not have accurately reflected the status of the participant. However, a random misclassification of this nature would tend to underestimate the magnitude of trends, and therefore the actual association might be closer than that identified in the present study.

## Methods

### Research design and participants

The ISSA-CKD (Iki City Epidemiological Study of Atherosclerosis and Chronic Kidney Disease) study is a retrospective study of the residents of Iki City, Japan. The details of this study have been provided previously^24–28^. Iki City has approximately 26,000 residents and is on an island in the northern part of Nagasaki Prefecture. A total of 8024 residents (≥ 30 years old) who underwent an annual health examination in Iki City at least once between 2008 and 2019 were eligible for inclusion in the present study. After excluding 1,902 people for whom less than a year of follow-up data were available, 544 who had hyperuricemia at baseline, and 11 with missing waist circumference data, 5567 residents (2331 men and 3236 women) remained for inclusion in the analysis. An opt-out approach was used to obtain the informed consent of the participants and the study was approved by the Fukuoka University Medical Ethics Review Board (No.2017M010). The study was conducted in accordance with the principles of the Declaration of Helsinki and the Ethical Principles for Medical Research and Ethical Guidelines for Medical and Biological Research Involving Human Subjects of the Japanese government.

### Data collection

At each health check, each participant’s waist circumference was measured in the standing position during standard expiration, at the umbilical level, as per the guidelines of Japan’s Ministry of Health, Labour and Welfare, by trained medical staff. The participants were placed into sex-specific groups according to tertile of waist circumference, because of the heterogeneous distribution of waist circumference in men and women.

BMI (kg/m^2^) was calculated as body mass (kg)/height (m)^2^, and a BMI ≥ 25 kg/m^2^ was regarded as indicating obesity^29^. After at least 5 min of rest in the sitting position, each participant’s blood pressure (BP) was measured twice using mercury, anaeroid or automated sphygmomanometers with appropriate-sized cuffs placed on their right upper arm. The mean of two BP values was used in the present analysis. A BP of ≥ 140/90 mmHg or the use of BP-lowering medication was regarded as indicating hypertension.

A standard questionnaire was used to obtain information regarding the participants’ smoking habits, alcohol consumption, and exercise status at baseline. Participants were classified as current smokers (regular smokers for ≥ 6 months) or not. Alcohol consumption was classified into whether or not the participant reported current daily drinking. Exercise status was classified as regular exercise (≥ 30 min per day at least twice weekly) or no regular exercise.

Fasting or non-fasting samples of blood and urine were obtained from each participant. The serum uric acid concentration was measured using an enzymatic method. High-performance liquid chromatography was used to measure glycated haemoglobin (HbA1c) levels (National Glycohemoglobin Standardization Program value). An enzymatic method was used to measure plasma glucose concentration. HbA1c ≥ 6.5%, fasting blood glucose ≥ 7.0 mmol/L, non-fasting blood glucose ≥ 11.1 mmol/L or the use of glucose-lowering therapies was regarded as indicating the presence of diabetes. The serum triglyceride, high-density lipoprotein (HDL)-cholesterol and low-density lipoprotein (LDL)-cholesterol concentrations were measured using enzymatic methods. An LDL cholesterol ≥ 3.62 mmol/l, HDL cholesterol < 1.03 mmol/l, triglycerides ≥ 1.69 mmol/l or the use of lipid-lowering medication was regarded as indicating dyslipidaemia. An enzymatic method was used to measure the serum creatinine concentration. The estimated glomerular filtration rate (eGFR) was calculated as follows: eGFR (mL/min/1.73 m^2^) = 194 × serum creatinine (mg/dL)^−1.094^ × age^−0.287^ (× 0.739 if female)^30^. Urinary protein concentration was assessed using a dipstick, and proteinuria was defined as a score of (1 +) or higher. eGFR < 60 mL/min/1.73 m^2^ and/or proteinuria was regarded as indicating the presence of CKD.

### Definition of the outcome

The outcome was the incidence of hyperuricemia (uric acid concentration > 7.0 mg/dL [416 µmol/L])^1^ during follow-up.

### Statistical analysis

SAS version 9.4 (Cary, NC, USA) was used for all the statistical analyses. Tertile groups of waist circumference were defined separately for men and women using the “Rank” procedure of SAS. Continuous data are presented as the mean ± SD, and trends across the tertile groups were evaluated using a simple regression model. Categorical data are presented as frequencies and percentages, and trends across the tertile groups were evaluated using a logistic regression model. The person-year approach was used to estimate incidences of hyperuricemia in the sample as a whole and in the tertile groups. The relationship between waist circumference and the incidence of hyperuricemia was evaluated using Cox proportional hazards models. Mediation analysis was performed according to a method described by Discacciati et al.^31^.

The effects of waist circumference on the incidence of hyperuricemia were compared between subgroups defined according to age; the presence or absence of hypertension, diabetes, dyslipidaemia, and CKD; smoking habits; alcohol consumption habits; and exercise habits by adding interaction terms to the statistical models. All the tests were two-tailed. *P* < 0.05 was considered to represent statistical significance.

## Conclusion

In the present longitudinal study of the general Japanese population, people with higher waist circumference were found to be at a higher risk of subsequent hyperuricemia, whether men or women. These results suggest that lifestyle interventions for individuals with abdominal obesity could prevent the development of hyperuricemia.

## Data Availability

The datasets generated and analysed within the current study are not publicly available as they are the property of Iki City, Nagasaki, Japan. Their publication would be in violation of the Act on the Protection of Personal Information of the Japanese government but are available from the corresponding author on reasonable request**.**
